# Accumulation of eosinophils in intestine-draining mesenteric lymph nodes occurs after *Trichuris muris* infection

**DOI:** 10.1111/j.1365-3024.2010.01246.x

**Published:** 2011-01

**Authors:** M SVENSSON, L BELL, M C LITTLE, M DeSCHOOLMEESTER, R M LOCKSLEY, K J ELSE

**Affiliations:** 1 Faculty of Life Sciences, University of ManchesterManchester, UK; 2 Department of Medicine, Microbiology and Immunology, Howard Hughes Medical Institute, University of California San FranciscoSan Francisco, CA, USA

**Keywords:** eosinophils, IL-4, large intestine, mesenteric lymph nodes, *Trichuris muris*

## Abstract

Eosinophils have recently been demonstrated capable of localizing to lymph nodes that drain mucosal surfaces, in particular during T helper 2 (Th2) responses. Resistance of mice to infection with the gastrointestinal nematode *Trichuris muris* depends critically on mounting of a Th2 response and represents a useful model system to investigate Th2 responses. Following infection of resistant BALB/c mice with *T. muris*, we observed accumulation of eosinophils in intestine-draining mesenteric lymph nodes (MLNs). The accumulation of MLN eosinophils was initiated during the second week of infection and peaked during worm expulsion. In contrast, we detected a comparably late and modest increase in eosinophil numbers in the MLNs of infected susceptible AKR mice. MLN eosinophils localized preferentially to the medullary region of the lymph node, displayed an activated phenotype and contributed to the interleukin-4 (IL-4) response in the MLN. Despite this, mice genetically deficient in eosinophils efficiently generated IL-4-expressing CD4^+^ T cells, produced Th2 cytokines and mediated worm expulsion during primary *T. muris* infection. Thus, IL-4-expressing eosinophils accumulate in MLNs of *T. muris-*infected BALB/c mice but are dispensable for worm expulsion and generation of Th2 responses, suggesting a distinct or subtle role of MLN eosinophils in the immune response to *T. muris* infection.

## Introduction

Eosinophils are innate immune cells associated with T helper 2 (Th2) immune responses. In mice, eosinophils also comprise a constitutive part of the innate cell repertoire of the intestine (1,2). Eosinophils accumulate in affected tissues in response to most nematode infections and in the intestine and airways during inflammatory disorders of the gastrointestinal tract (e.g. inflammatory bowel diseases) and airways (e.g. asthma and allergic responses), respectively. The function of eosinophils during these diseases has, however, mainly been associated with the tissue destruction that is a hallmark of these disorders and in which eosinophils are thought to be involved. Thus, classically, eosinophils are considered to be end-stage effector cells, with an effector mechanism predominantly confined to release of their cytotoxic granule content (3). Recent reports have demonstrated that eosinophils can also be detected in mucosal-draining lymph nodes during mucosal inflammation caused by infection with *Nippostrongyles brasiliensis* or in an airways inflammation model (4,5). Tissue eosinophils can express molecules involved during lymphocyte activation (e.g. major histocompatibility (MHC)-II and CD80) (6–8), suggesting that they may be directly or indirectly involved in adaptive immune responses (9,10). However, whether lymph node eosinophils express MHC-II and co-stimulatory molecules and their function remains largely uncharacterized.

Infections of humans with gastrointestinal nematodes, e.g. *Trichuris trichiura*, represent some of the most prevalent diseases globally, with an estimated 3·5 billion people harbouring at least one gastrointestinal nematode (11). *Trichuris muris* infection in the mouse serves as a good model of human intestinal nematode infection and, at a more fundamental level, represents a valuable tool to dissect the factors that combine to promote the efficient generation and execution of polarized Th immune responses. CD4^+^ Th cells play a central role in the immune response to *T. muris* infection (12,13), and the nature of the Th response is of critical importance. Mesenteric lymph node (MLN) cells from Th2-prone mouse strains, including BALB/c mice, produce Th2 cytokines (IL-4, IL-5, IL-9 and IL-13) in response to *T. muris* infection. The Th2 response causes expulsion of worms between day 14 (d14) and d19p.i. in BALB/c mice, with the time point of expulsion varying in other resistant mouse strains according to genetic background and MHC haplotype (14,15). Expulsion is mediated by a mechanism at least partially involving increased intestinal epithelial turnover, regulated by IL-13 (16). In contrast, Th1-prone mouse strains, e.g. AKR mice, fail to expel worms and develop chronic infections (17).

In the current study, we have identified and characterized a population of eosinophils that accumulate in the MLNs of Th2-dominated resistant mouse strains in response to *T. muris* infection.

## Materials and methods

### Mice

Male BALB/c and AKR mice were purchased from Harlan UK. ΔdblGATA-1 mice, a kind gift from Professor Stuart Orkin and previously described (18), were on a 4get mouse (19) background, as previously described (5,20). To generate wild-type 4get mice, we crossed male ΔdblGATA-1×4get mice with female BALB/c mice and used the F1 offspring in experiments. All mice were maintained in microisolator cages in the animal facility at the University of Manchester. Mice used in experiments were 6- to 12-week-old male mice. All animal work was performed under the regulations of the Home Office Scientific Procedures Act (1986).

### Parasites

The maintenance of the *T. muris* parasite and methods used for infection and large intestinal worm burden assessment were as previously described (15,21). Mice were infected by oral gavage with 120–150 infective eggs.

### Antibodies and reagents for flow cytometry

The following antibodies were used in this study: fluorescein isothiocyanate (FITC)-conjugated anti-CD4 (clone GK1·5), anti-CD8β (53–5·8), anti-Gr-1 (RB6-8C5), anti-B220 (RA3-6B2), anti-CD49b (DX5), anti-CD11b (M1/70), and rat IgG2a (RG7/1·30), rat IgG2b (A95-1), rat IgM (R4-22) and rat IgG1 (R3-34) isotype controls, phycoerythrin (PE)-conjugated anti-Ly6G (1A8), anti-CD8α (53–6·7), anti-Siglec-F (E50-2440), anti-CD86 (GL-1), and rat IgG2a (R35-95) isotype control, allophycocyanin (APC)-conjugated anti-CD11c (HL3) and anti-c-kit (2B8), and rat IgG2a (R35-95) and rat IgG2b (A95-1) isotype controls, and biotinylated rat IgG2a (R35-95) and rat IgG2b (A95-1) isotype controls were all from BD Biosciences (Oxford, UK). Unconjugated anti-CD16/32 (93), PE-conjugated anti-FcεR I (MAR-1) and syrian hamster IgG isotype control, biotinylated anti-MHC-II (M5/114·15·2), anti-CD80 (16-10A1), anti-CD40 (1C10) and armenian hamster IgG (eBio299Arm) isotype control, APC-conjugated anti-F4/80 (BM8) and anti-CD62L (MEL-14), and FITC- and APC-conjugated streptavidin were from eBiosciences (Insight Biotechnology, Wembley, UK). PE-conjugated anti-CCR3 (83101) was from R’n’D systems, and 7-amino-actinomycin D (7AAD) was from Sigma-Aldrich (Gillingham, UK).

### Cell isolations

Mesenteric lymph nodes and peripheral lymph nodes (PLN; superficial inguinal) were excised, single cell suspensions prepared by crushing organs through 70 -μm cell strainers (BD Biosciences) and cells washed in fluorescence-activated cell sorting (FACS) buffer (phosphate buffered saline (PBS) supplemented with 2% FCS (PAA Laboratories, Pasching, Austria) and 0·05% sodium azide (Sigma-Aldrich)). Bone marrow cells were acquired from femurs by cutting the bone ends and flushing with 5 mL of FACS buffer using a 23-G needle. Blood was collected by cardiac puncture. For the isolation of large intestinal lamina propria cells, the caecum and first 5 cm of the proximal colon were collected. The tissue was rinsed and cut into 5 -mm pieces. To remove the epithelial layer, tissue pieces were incubated sequentially in HBSS (PAA) supplemented with 2% FCS and 1 mm EDTA (Sigma-Aldrich) and 10 mm or 2 mm DTT (Sigma-Aldrich), respectively, for 20 min each. Remaining tissue was digested in RPMI (PAA) supplemented with 5% FCS, 2 mm l-glutamine (Invitrogen, Paisley, UK), 1 mg/mL collagenase V (Sigma-Aldrich) and 1 mg/mL collagenase D (Roche diagnostics, Burgess Hill, UK) for 1 h. Resulting cells were washed and leucocytes enriched by Percoll gradient centrifugation (40/70).

### Flow cytometry and FACS sorting

Flow cytometry was performed as previously described (22). Data were acquired on a FACSCalibur (BD Biosciences) and analysed using FloJo Software (Treestar Inc., Ashland, OR, USA). For FACS sorting of eosinophils, MLN cells were incubated with antibodies towards CD11b, F4/80 and Siglec-F as described earlier and sorted on a FACSAria (BD Biosciences). Sorted cells were >96% pure (data not shown).

### Cytospin

FACS-sorted cells (1 × 10^5^) were spun onto gelatin-coated slides. Following fixation with cold 4% paraformaldehyde, cells were stained with H&E.

### Immunofluorescence microscopy

Mesenteric lymph node tissue frozen in OCT (R.A. Lamb, Thermo Scientific, Loughborough, UK) was sectioned into 6- μm-thick sections on a Microm HM560 cryostat (Microm, Bicester, UK). Tissue was fixed in cold 4% paraformaldehyde and sequentially blocked with Renaissance Blocking reagent (Perkin-Elmer), 10% normal mouse serum (Sigma-Aldrich) and avidin/biotin (Vector Laboratories, Peterborough, UK). Tissues were stained with rat IgG2a anti-Siglec-F (5 μg/mL; clone E50-2440; BD Biosciences), followed by mouse anti-rat IgG2a biotin (5 μg/mL; RG7/1·30; BD biosciences). Next, tissue was incubated with ABC kit (Vector Laboratories), followed by Cy3-conjugated tyramide (Perkin-Elmer, Cambridge, UK), all according to manufacturers’ instructions. To co-stain with B-cell markers and inter-cellular adhesion molecule-1 (ICAM-1), tissue was again blocked as described earlier plus with 50 μg/mL rat IgG (Sigma-Aldrich), and incubated with 5 μg/mL biotinylated anti-B220 (RA3-6B2; BD biosciences), or rat IgG2a anti-ICAM-1 (2·5 μg/mL; KAT-1; eBiosciences, Hatfield, UK) followed by mouse anti-rat IgG2a biotin (5 μg/mL; RG7/1·30; BD Biosciences). Next, tissue was stained with 30 μg/mL fluorescein-conjugated avidin D (Vector Laboratories). Slides were washed twice with PBS supplemented with 0·05% BSA (PAA) between each step. Finally, slides were mounted in Vector Shield containing DAPI (Vector Laboratories).

### Statistical tests

Statistical testing was performed using Student’s *t*-test for two-parameter comparisons and anova with Tukey’s post-test for multiparameter comparisons. Statistical comparisons were performed using GraphPad PRISM software (GraphPad Software, La Jolla, CA, USA).

## Results

### Trichuris muris infection leads to accumulation of eosinophils in the MLN

**Figure 1:**
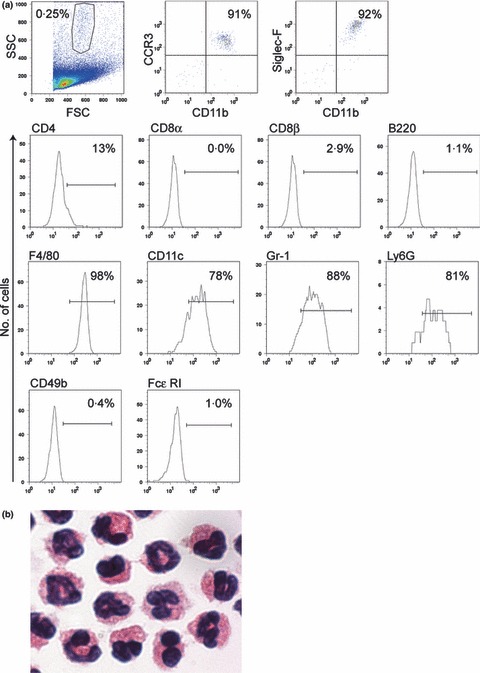
CCR3^+^ and Siglec-F^+^ eosinophils accumulate in mesenteric lymph nodes (MLNs) of *T. muris-*infected BALB/c mice. (a) Lineage marker expression by granular cells in the MLNs of *T. muris*-infected BALB/c mice. MLN cells isolated from *T. muris*-infected BALB/c mice at d21p.i. were analysed by flow cytometry to identify a population of SSC^hi^ cells accumulating in response to infection. The upper left panel shows the forward and side-scatter profile used to gate out the cell population of interest, and the remaining graphs show staining with various lineage markers following gating on live (7AAD negative) cells (dot plots) and live CD11b^+^ Siglec-F^+^ or live CD11b^+^ F4/80^+^ cells (histograms). Gates have been set according to staining with isotype control antibodies. Data are from individual mice from one representative experiment of 4–7 performed. (b) SSC^hi^ CD11b^+^ F4/80^+^ Siglec-F^+^ cells isolated from MLNs are eosinophils. SSC^hi^ CD11b^+^ F4/80^+^ Siglec-F^+^ cells were FACS-sorted from MLNs collected from BALB/c mice at d21p.i. The purified cells were cytospun onto slides and stained with H&E to confirm their identity as eosinophils.

To identify cells potentially involved during the protective immune response towards *T. muris*, we characterized the cellular composition of the intestine-draining MLNs after infection of resistant BALB/c mice with *T. muris*. Interestingly, we observed accumulation of a population of highly granular cells (side scatter^hi^; SSC^hi^) in the MLNs (Figure 1a, upper left panel). The cells were further characterized by flow cytometry for the expression of various markers associated with granular cells, such as Siglec-F and the chemokine receptor CCR3 (eosinophils) (23), Ly6G and Gr-1 (neutrophils), CD49b and FcεR I (basophils and mast cells), as well as other lineage-specific surface receptors. Strikingly, more than 90% of the SSC^hi^ cells expressed Siglec-F and CCR3 together with the integrin CD11b, indicating that they were eosinophils (Figure 1a). In contrast, the SSC^hi^ cells were negative for markers associated with T and B cells, basophils and mast cells but expressed some markers associated with, but not exclusively expressed by, dendritic cells (DCs) (CD11c), macrophages (F4/80) and neutrophils (Ly6G and Gr-1) (Figure 1a). The SSC^hi^ population was FACS-sorted from MLNs of BALB/c mice 21 days after *T. muris* infection based on their size and granularity profile, viability and surface expression of CD11b, F4/80 and Siglec-F. The sorted cells were cytospun and stained with H&E. In agreement with their expression of CCR3 and Siglec-F, the sorted cells uniformly stained positive with eosin, confirming that they were eosinophils (Figure 1b). Thus, infection with *T. muris* leads to the accumulation of eosinophils in MLNs.

### Eosinophils accumulate preferentially in MLNs of *T. muris*-resistant BALB/c mice

**Figure 2:**
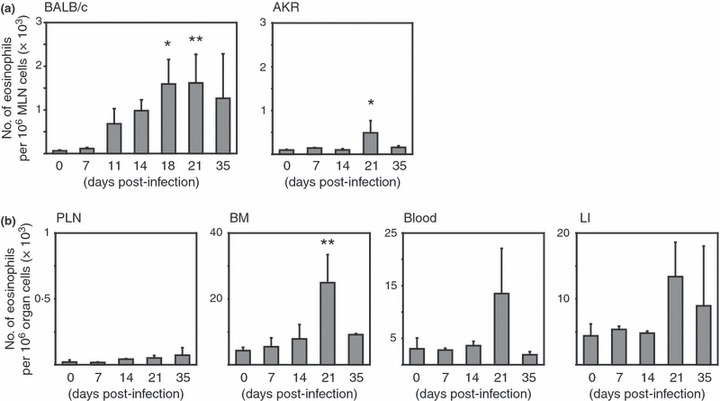
Eosinophil accumulation as a function of time, strain and tissue in *T. muris-*infected mice. Eosinophil accumulation in (a) mesenteric lymph nodes (MLNs) and (b) PLNs, blood, bone marrow and the large intestinal lamina propria, at various time points after infection of (a and b) BALB/c and (a) AKR mice. At various time points after infection, tissues were collected and examined for the presence of eosinophils by flow cytometry, by gating on live SSC^hi^ CD11b^+^ F4/80^+^ Siglec-F^+^ cells. (a) Bars show the mean number of eosinophils per 10^6^ total MLN cells ± SEM. Results are from 6 to 28 mice from 3 to 9 experiments (BALB/c) and four mice from two experiments (AKR), respectively. Statistical tests compare the number of eosinophils post-infection to the numbers seen in uninfected animals (d0). Statistical analysis was performed using anova, resulting in an overall *P*-value of *P* < 0·0039. Tukey’s post-test was used to compare individual time points, resulting in significant differences as displayed in the graph. *P < 0·05; **P < 0·01. (b) Bars show the mean numbers of eosinophils per 10^6^ total tissue cells ± SEM. Results are from 3 to 9 mice from 1 to 3 experiments. Statistical tests compare the number of eosinophils post-infection to the numbers seen in uninfected animals (d0). Statistical analysis was carried out using anova, with an overall *P* -value of *P* < 0·0016. Tukey’s post-test was used to compare individual time points, resulting in significant differences as displayed in the graph. **P < 0·01.

To further determine the kinetics of and requirements for eosinophil accumulation, we infected BALB/c (resistant to *T. muris*) and AKR (susceptible) mice with *T. muris* and analysed the presence of eosinophils in MLNs at various time points post-infection. In BALB/c mice, increased numbers of MLN eosinophils were first detected at d11p.i. (Figure 2a). The number of MLN eosinophils peaked at around d18 to d21p.i., thereafter numbers declined (Figure 2a). In contrast, there was no accumulation of eosinophils in the MLNs of susceptible AKR mice during the first 2 weeks of infection (Figure 2a). However, despite their predisposition to mount a Th1 response post-infection, AKR mice had significantly increased numbers of eosinophils in the MLNs at d21p.i. (Figure 2a), although the number of eosinophils detected was modest compared to that in BALB/c mice (AKR: 5-fold increase compared to naïve levels; BALB/c: 26-fold increase compared to naïve levels). Further, in contrast to MLNs, the number of eosinophils detected in PLNs remained low throughout infection in BALB/c mice (Figure 2b). In the bone marrow, there was a significant increase in the number of eosinophils at d21p.i. (Figure 2b). This increase was mirrored in the blood and at the site of infection (large intestine); however, these increases were not significant (Figure 2b). Thus, increased numbers of eosinophils can be detected from d11p.i. in the MLNs of resistant mice. In the bone marrow, blood and large intestinal mucosa of *T. muris*-infected BALB/c mice and in MLNs of susceptible AKR mice, eosinophil numbers are increased at d21p.i.

### MLN eosinophils localize to the lymph node medulla and interfollicular region

**Figure 3:**
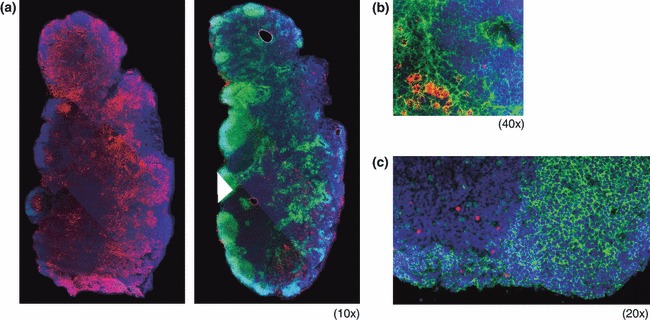
Mesenteric lymph node (MLN) eosinophils localize to the lymph node medulla. Intranodal localization of MLN eosinophils. Immunofluorescence analysis of MLN tissue collected from *T. muris-*infected BALB/c mice at d18p.i. Tissue was stained with (A, left panel) ICAM-1 (red) and DAPI (blue), (A, right panel) B220 (green), Siglec-F (red) and DAPI (blue), (b) ICAM-1 (green), Siglec-F (red) and DAPI (blue), and (c) Siglec-F (red), B220 (green) and DAPI (blue). Magnifications are as stated in the figure. Images were collected on an Olympus BX51 upright microscope using a 20×/0·50 Plan Fln objective and captured using a Coolsnap ES camera (Photometrics) through Meta Vue Software (Molecular Devices). Specific band-pass filter sets for DAPI, FITC and Cy-3 were used to prevent bleed-through from one channel to the next. Images were processed and analysed using Adobe Photoshop (Adobe). The whole-LN views in (a) are mosaics generated of multiple individual images.

Next, we were interested in determining the intranodal location of the MLN eosinophils. MLN tissue from BALB/c mice at the peak of eosinophil accumulation (d18p.i.) was sectioned and labelled with fluorochrome-conjugated antibodies specific for Siglec-F, B220 and ICAM-1 to identify eosinophils, B-cell follicles and medullary cords (24), respectively, and analysed by immunofluorescence microscopy. The majority of MLN eosinophils localized to areas rich in ICAM-1^+^ medullary cords (Figure 3a, b). In addition, eosinophils were also detected in the subcapsular sinus (Figure 3a) and T-cell area (Figure 3c), while in contrast Siglec-F-positive cells were not detected within B-cell follicles (Figure 3c). Thus, MLN eosinophils localize preferentially to the medullary region of the lymph node.

### MLN eosinophils display an activated phenotype and contribute to the IL-4 response in the MLN

**Figure 4:**
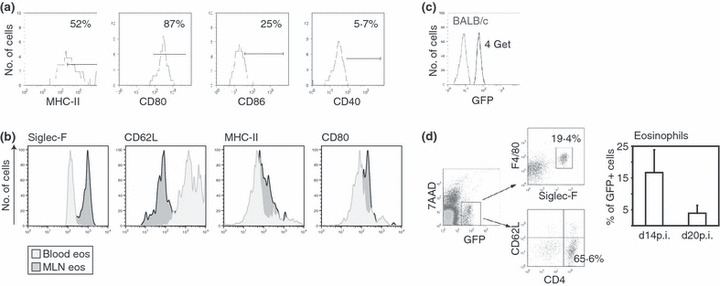
Mesenteric lymph node (MLN) eosinophils display an activated phenotype and contribute to the IL-4 response in the MLN*.* MLN eosinophils were isolated from (a and c) BALB/c and (b to d) 4get mice at d21p.i. and examined by flow cytometry. (a) Expression of molecules associated with interaction with T cells. Graphs show histograms for each marker following gating on live SSC^hi^ CD11b^+^ F4/80^+^ or SSC^hi^ CD11b^+^ Siglec-F^+^ cells. Data are from individual mice from one representative experiment of 3–5 performed. (b) MLN eosinophils display an activated phenotype. MLN and blood eosinophils isolated from 4get mice at d20p.i. were examined by flow cytometry for the expression of Siglec-F, CD62L, MHC-II and CD80. Histograms show overlain MLN (dark grey) and blood (light grey) eosinophils after gating on live Siglec-F^+^ GFP^+^ cells. (C and D) MLN eosinophils constitutively express IL-4 and contribute to the MLN IL-4 response. BALB/c and 4get mice were infected with *T. muris*. At d14 and d20p.i., MLN cells were isolated and analysed by flow cytometry. (c) GFP expression in eosinophils (histograms are shown following gating on live Siglec-F^+^ F4/80^+^ cells) isolated at d14p.i. from the MLN of 4get (black line) and BALB/c (grey line) mice. Data are from one experiment of two performed, with ten to eleven individually analysed mice per group and time point. (d) Identity of GFP^+^ cells in MLN following *T. muris* infection of 4get mice. MLN cells isolated from 4get mice at d14 and d20p.i. were gated on live GFP^+^ cells and analysed for the expression of markers identifying eosinophils and activated CD4^+^ T cells. Flow cytometry plots show MLN cells from 4get mice at d14p.i., and numbers denote the percentages of cells within the respective gates. Bar graph shows the mean ± SD of the percentage of eosinophils among GFP^+^ cells. Data are combined from five to nine individually analysed mice from two experiments.

To determine whether MLN eosinophils express molecules that would allow them to directly interact with and activate T cells, we determined the expression of MHC-II, CD40, CD80 and CD86 on eosinophils purified from MLNs of *T. muris*-infected BALB/c mice at d14 and d21p.i. (Figure 4a, and data not shown). MHC-II was expressed on 60·1 ± 13·2% (mean ± SD, *n* = 5) of MLN eosinophils at d21p.i. (Figure 4a). The level of MHC-II expressed by MLN eosinophils was lower compared to that on MLN DCs (data not shown). Of the co-stimulatory molecules, CD80 was expressed on the majority of MLN eosinophils (90·7 ± 3·6% (mean ± SD, *n* = 3), while CD86 and CD40 were expressed only by a minority of cells (CD86: 27·7 ± 3·9 (mean ± SD, *n* = 3); CD40: 2·8 ± 0·5 (mean ± SD, *n* = 3) (Figure 4a). Expression of MHC-II and co-stimulatory molecules was similar at d14 and d21p.i. (data not shown).

To investigate whether MLN eosinophils displayed an activated phenotype, we compared their expression of markers known to be regulated in an activation status-dependent manner to that of blood eosinophils from the same mice. Interestingly, MLN eosinophils expressed higher levels of Siglec-F than blood eosinophils (Figure 4b), a characteristic typical of activated eosinophils (5). MLN eosinophils also expressed higher levels of MHC-II and CD80 than blood eosinophils and had downregulated CD62L (Figure 4b), which is also typical of activated eosinophils (25).

Finally, to determine whether the MLN eosinophils expressed IL-4 following *T. muris* infection, we utilized 4get mice that have the reporter green fluorescent protein (GFP) linked to the IL-4 promoter (19). Using this strain, it has previously been demonstrated that bone marrow, blood and lung eosinophils constitutively transcribe IL-4 (25,26). Initially, we confirmed that eosinophils accumulate in the MLNs of *T. muris*-infected 4get mice in a similar manner as observed in BALB/c mice following infection (data not shown). To investigate whether MLN eosinophils were GFP^+^ in 4get mice and therefore actively transcribing the IL-4 gene, we phenotyped MLN eosinophils at d14 and d20 post-*T. muris* infection. Eosinophils in MLNs were homogenously GFP^+^ at both d14 and d20p.i. (Figure 4c, and data not shown). To further characterize the IL-4-expressing GFP^+^ cells in the MLN and to determine the frequency of eosinophils among total GFP^+^ cells, we examined the expression of various lineage markers on GFP^+^ cells in MLNs of *T. muris*-infected 4get mice (Figure 4d). Eosinophils constituted 15–20% of the GFP^+^ MLN cells at d14p.i. (Figure 4d). Besides eosinophils, CD4^+^ CD62L^low^ T cells were the largely dominating GFP^+^ population in MLNs of 4get mice, constituting 65–70% of GFP^+^ MLN cells at d14p.i. (Figure 4d). At d20p.i., the percentage of eosinophils among GFP^+^ cells had decreased (Figure 4d). In addition to eosinophils and CD4^+^ CD62L^low^ T cells, the GFP^+^ population in 4get mice contained CD4^+^ CD62L^hi^ T cells (approximately 5% of GFP^+^ cells) and small populations (<3%) of DCs, basophils and mast cells (data not shown).

Taken together, these results demonstrate that MLN eosinophils display an activated phenotype and contribute to the IL-4 response generated in the MLN following *T. muris* infection.

### MLN eosinophils are not required for production of Th2 cytokines or expulsion of worms

**Figure 5:**
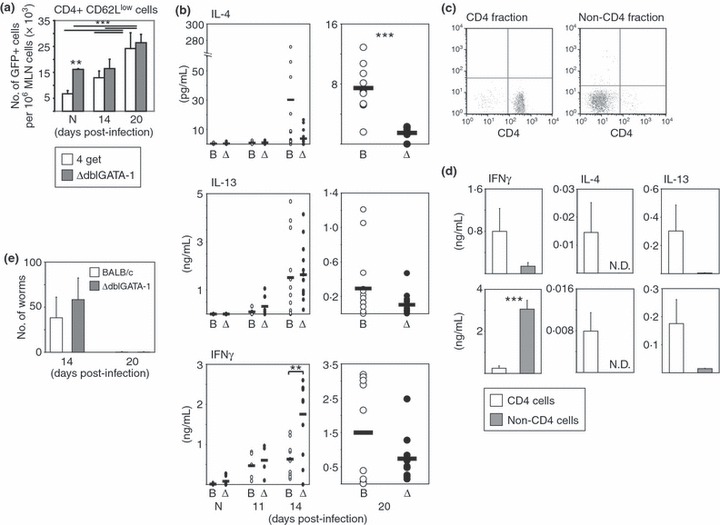
Cytokine production and worm expulsion in BALB/c and ΔdblGATA-1 mice following *T. muris* infection. (a) Eosinophil-deficient mice have normal numbers of IL-4-expressing CD4^+^ T cells. Mesenteric lymph node (MLN) cells were isolated from 4get and ΔdblGATA-1 mice prior to and at d14 and 20 post-*T. muris* infection. Expression of GFP was determined on CD4^+^ CD62L^low^ T cells, and the mean ± SD number of GFP^+^ CD4^+^ CD62L^low^ T cells per 10^6^ total MLN cells was calculated. Data are combined from four to nine individually analysed mice from two experiments. Statistical analysis was performed using anova, with an overall *P*-value of *P* ≤ 0·0001. Comparisons between individual groups were performed using Tukey’s post-test and are displayed in the graph. **P ≤ 0·01; ***P ≤ 0·001. N, naïve. (b) Eosinophil-deficient mice produce equal levels of Th2 cytokines compared to wild-type mice at the initiation and peak of the anti-*T. muris* response. BALB/c and ΔdblGATA-1 mice were infected with *T. muris*. Prior to infection and at days 11, 14 and 20 post-infection, MLN cells were isolated and re-stimulated *in vitro* with 50 μg/mL E/S antigen for 48 h. Cell culture supernatants were collected and the IL-4, IL-13 and IFNγ contents determined by CBA analysis. Each dot (clear dots: BALB/c; filled dots: ΔdblGATA-1) in the graph represents one individual mouse, and the line represents the mean. Graphs show nine to sixteen individual mice from three separate experiments, except for IFNγ which show 4–12 mice from two experiments. Statistical tests compare differences between BALB/c and ΔdblGATA-1 mice at each time point. **P ≤ 0·01; ***P ≤ 0·001 (Student’s *t*-test). N, naïve; B, BALB/c mice; Δ, ΔdblGATA-1 mice. (C and D) Th2 cytokines are CD4^+^ T cell-derived. CD4^+^ T and non-CD4^+^ cells were isolated from BALB/c mice at d20p.i. by MACS. Purified cells were polyclonally re-stimulated with anti-CD3 or con A for 48 h. (c) The purity of the sorted CD4^+^ T cells and non-CD4 cells was analysed by flow cytometry. Data are from one representative separation of five performed. (d) Cell culture supernatants were collected 48 h after re-stimulation with anti-CD3 (top panel) or conA (bottom panel) and the content of IFNγ, IL-4 and IL-13 determined by CBA analysis. Bars show mean ± SEM of the cytokine levels from one experiment (*n* = 5). *P ≤ 0·05; ***P ≤ 0·001 (Student’s *t*-test). No statistical testing could be performed for IL-4, as no IL-4 was detected in supernatants from non-CD4 cells. N.D., not detected. (e) Worm burden assessment in the large intestine of BALB/c and ΔdblGATA-1 mice. BALB/c and ΔdblGATA-1 mice were infected with *T. muris*. At d14 and 20p.i., large intestines were collected and the number of worms counted. Bars show the mean number of worms ± SEM from five to six mice. Results are from one experiment of five performed.

A potential role of large intestinal eosinophils in protective immunity against *T. muris* has been previously investigated (27,28). In these studies, an absence of eosinophils did not alter worm expulsion. However, neither study took into account the MLN eosinophils, and both models were compromised in that the eosinophil deficiency was not complete. Therefore, to examine a potential role of eosinophil in the immune response to *T. muris* infection, we utilized ΔdblGATA-1 mice, which are genetically deficient in eosinophils because of the deletion of a high-affinity binding site for GATA-1 in the GATA-1 promoter (18). We first examined the generation of IL-4-expressing CD4^+^ CD62L^low^ T cells following *T. muris* infection in the presence or absence of eosinophils, by determining the number of GFP^+^ CD4^+^ CD62L^low^ T cells per 10^6^ MLN cells in ΔdblGATA-1 and 4get mice following *T. muris* infection. Prior to infection, MLNs of ΔdblGATA-1 mice contained significantly more GFP^+^ CD4^+^ CD62L^low^ T cells compared to 4get MLNs (Figure 5a). The GFP^+^ CD4^+^ CD62L^low^ T cells increased significantly in both strains in response to infection, but despite the lack of eosinophils in ΔdblGATA-1 mice, we detected equal numbers of GFP^+^ CD4^+^ CD62L^low^ T cells per 10^6^ MLN cells in 4get and ΔdblGATA-1 mice at both d14 and d20 post-*T. muris* infection (Figure 5a). Thus, eosinophils are not required for the generation of IL-4-expressing CD4^+^ T cells.

Given that the expulsion of *T. muris* is critically dependent on a Th2 response and MLN eosinophils express high levels of IL-4, we next investigated whether eosinophils influenced the Th2 cytokine production in the MLN of *T. muris*-infected mice. Before and at days 11, 14, 16, 18 and 20 after *T. muris* infection of BALB/c and ΔdblGATA-1 mice, MLN cells were isolated, re-stimulated with *T. muris*-derived E/S antigen and the cytokine content in cell culture supernatants determined after 48 h (Figure 5b, and data not shown). Production of the Th2 cytokines IL-4, IL-5 and IL-13 followed a similar kinetic between BALB/c-derived and ΔdblGATA-1-derived MLN cells, with a peak occurring at d14p.i. (Figure 5b, and data not shown). At d20p.i., significantly more IL-4 was produced in cultures with MLN cells from BALB/c compared to ΔdblGATA-1 mice (Figure 5b). For interferon-gamma (IFNγ), a slightly faster kinetic of production was observed in ΔdblGATA-1 mice, with significantly more IFNγ being produced by ΔdblGATA-1-derived MLN cells at d14p.i., while there were no significant differences in IFNγ production at later time points (Figure 5b). Therefore, while there was no difference in the levels of Th2 cytokines produced between BALB/c and ΔdblGATA-1 mice during the initiation and peak of the immune response, BALB/c-derived MLN cells produced significantly more IL-4 at the late stage of the response. To confirm that the cytokines produced derived from CD4^+^ T cells and not from other cells in the cultures, we sorted total MLN cells from d20p.i. BALB/c mice into CD4^+^ and non-CD4 cells (Figure 5c), followed by *in vitro* re-stimulation. Because no antigen-presenting cells are present in the CD4^+^ fraction, cells were polyclonally activated with anti-CD3 or con A instead of E/S antigen. Following 48h of stimulation, supernatants were harvested and the cytokine contents examined. In response to anti-CD3 stimulation, CD4^+^ T cells produced all of the cytokines examined, while in contrast low levels or no cytokine was produced by non-CD4 cells (Figure 5d, top). After con A-stimulation, CD4^+^, but not non-CD4 cells, produced the Th2 cytokines IL-4 and IL-13. In contrast, con A-stimulated non-CD4 cells produced more IFNγ compared to CD4^+^ cells (Figure 5d, bottom). Together, these results demonstrate that CD4^+^ T cells are the dominating source of Th2 cytokines after cytokines following *in vitro* re-stimulation of CD4^+^ MLN cells from *T. muris*-infected mice.

Finally, to determine whether the absence of eosinophils in these mice could lead to a delayed worm expulsion, we compared the worm burden in *T. muris*-infected BALB/c and ΔdblGATA-1 mice at d14 and d20p.i. We did not detect any significant differences in the worm burden between the mouse strains following analysis on d14 and d20p.i. (Figure 5e). Therefore, ΔdblGATA-1 mice expel worms with a normal kinetic following primary *T. muris* infection.

## Discussion

Several recent studies have demonstrated a previously unrecognized role of granulocytes (basophils and eosinophils) and mast cells as contributors to and regulators of adaptive immune responses in secondary lymphoid organs, with this role going beyond their prior classification as end-stage effector cells acting predominantly in infected tissues (7,10,29–32). In the current study, we have characterized a population of eosinophils detected in the draining MLNs of mice infected with the caecal-dwelling nematode *Trichuris muris*. A potential role of large intestinal eosinophils during primary infection with *T. muris* has previously been investigated (27,28). Both these studies revealed efficient worm expulsion despite reduced levels of eosinophils in the intestine. However, neither study examined the MLN eosinophils, and the eosinophil deficiency was not complete in these models unlike the ΔdblGATA-1 mice used in the current study.

Herein, we demonstrate that accumulation of MLN eosinophils during the first 2 weeks of *T. muris* infection occurs selectively in Th2-dominated resistant BALB/c mice, while in contrast there was only a modest increase in MLN eosinophil numbers occurring comparably late during infection of susceptible AKR mice. It is currently unknown what governs the increase in MLN eosinophils. Eosinophils develop in the bone marrow and leave as mature cells. In previous studies, eosinophil accumulation in various tissues has correlated with a decrease in bone marrow eosinophils, and it is therefore thought that accumulation is regulated by an increased release of mature eosinophils from the bone marrow rather than proliferation of peripheral eosinophils (33). Eosinophilia occurs as part of Th2 responses and the increased output and release of eosinophils from the bone marrow is regulated, at least partly, by IL-5 (34,35). In addition, eosinophils can be released from local tissues in response to increased levels of CCL11 in the circulation (36). IL-5 and CCL11 cooperate to generate an intestinal eosinophilia in a model of gastrointestinal allergy; here, initial eosinophil release from the bone marrow appears to be mediated by IL-5, whereas local production of CCL11 in the intestine mediates the recruitment from the blood to the intestine (33). Less is known, however, about the involvement of cytokines, chemokines and chemokine receptors during eosinophil migration to lymph nodes, although it has been demonstrated that CCR3 is dispensable for the migration of tracheally instilled eosinophils into draining paratracheal lymph nodes (7). In the current study, the output of eosinophils from the bone marrow increased in response to *T. muris* infection in BALB/c mice, as did eosinophil numbers in blood and the large intestine. However, eosinophil accumulation in MLNs occurred early, before the peak in IL-5 production by MLN cells (data not shown) and prior to the increased output of eosinophils from the bone marrow and increased levels of eosinophils in the blood and intestine. Surprisingly, eosinophil accumulation occurred also in the MLNs of Th1-prone AKR mice, albeit delayed and at reduced levels compared to Th2-prone BALB/c mice. Together this suggests that eosinophil accumulation in MLNs occurs at least in part independently of a Th2 response. Moreover, the numbers of eosinophils were sufficient in bone marrow, blood and large intestine even at steady state to support the observed increased number of MLN eosinophils. Therefore, we also cannot presently determine from which tissue eosinophils localize to the MLN, i.e. if this occurs via lymphatic vessel draining from the intestine or directly from the blood via the high endothelial venules. Further experimentation is required to clarify the regulation, route and underlying mechanism behind eosinophil accumulation in MLNs of *T. muris-*infected mice.

Eosinophils isolated from the MLN displayed an activated phenotype compared to blood eosinophils, characterized by high-level expression of Siglec-F and downregulated CD62L and higher expression of molecules involved during T-cell activation (MHC-II and CD80). Furthermore, upon analysis in 4get mice that have the reporter GFP coupled to the IL-4 promoter, MLN eosinophils were homogenously GFP^+^, demonstrating that they express the IL-4 gene. IL-4 is a key cytokine in adaptive anti-*T. muris* immune responses (17,37,38), although the source of early IL-4 and the signals that induce IL-4 production during *T. muris* infection is not known. In our experiments, eosinophils contributed to the IL-4 response in the MLN and indeed were the second largest IL-4-expressing population in the MLN at d14p.i. Despite this, normal numbers of IL-4-expressing CD4^+^ T cells were present in the MLN of *T. muris*-infected ΔdblGATA-1 mice, and similar levels of Th2 cytokines were produced at the early and peak of the primary response, and worm expulsion occurred with normal kinetic, demonstrating that eosinophils are not critical for the generation of a successful Th2 response to primary *T. muris* infection. Interestingly, significantly more IL-4 was produced by MLN cells from BALB/c mice compared to ΔdblGATA-1 mice during the resolution phase of the anti-*T. muris* response. Although we do not yet know the relevance of production of IL-4 at this stage of the immune response, it is plausible that a persistent cytokine response plays a role in, for example, tissue repair following expulsion or will be important in response to subsequent secondary infections. Eosinophils have previously been demonstrated to play a more prominent role during secondary infection (20). Furthermore, a recent report demonstrated that Gr-1^+^ IL-4-expressing eosinophils accumulated in the spleen following i.p. administration of alum and affected the secretion of IgM antibodies (32), demonstrating that secondary lymphoid organ eosinophils can also participate in B-cell antibody responses. Thus, further studies are required to examine a potential role of MLN eosinophils during the primary immune response to *T. muris* infection, and/or during challenge infection.

In conclusion, we demonstrate here that eosinophils localize to the MLNs of resistant BALB/c mice following infection with the gastrointestinal nematode *Trichuris muris*. MLN eosinophils display an activated phenotype and contribute to the IL-4 response in the MLN; however, generation of IL-4-expressing CD4^+^ T cells, Th2 cytokine production and worm expulsion occurred with a normal kinetic in eosinophil-deficient mice following primary *T. muris* infection, demonstrating that eosinophils were dispensable for the primary immune response to *T. muris* infection.
